# Liabilities of Patients

**Published:** 1890-12

**Authors:** 


LIABILITIES OF PATIENTS.—Mr. A. M. Hurlock, an attorney of Baltimore, is engaged in an attempt to get a bill through the Legislature of Maryland, which shall render a married woman's estate liable for medical services rendered to herself or to her children. Every now and then rich or well-to-do women with worthless husbands obtain under the present law the medical services of the doctor without compensation, and we think that a law such as proposed will materially help in stopping one of the leaks which wastes the stream of nutrition running into the lean professional purse. We trust that he may be successful, and that his example may prove contagious. The principal section of the proposed bill is as follows:

Section 1. *Be it enacted by the General Assembly of Maryland,* That married women shall be jointly liable with their husbands for medical services rendered to such married women or their children, that they may be sued jointly with their husbands for such services, and that judgments obtained in such suits may be a lien on their separate property.—*Med. News.*

